# Recent Advances in Polyamine Metabolism and Abiotic Stress Tolerance

**DOI:** 10.1155/2014/239621

**Published:** 2014-07-20

**Authors:** Parimalan Rangan, Rajkumar Subramani, Rajesh Kumar, Amit Kumar Singh, Rakesh Singh

**Affiliations:** Division of Genomic Resources, National Bureau of Plant Genetic Resources, New Delhi 110012, India

## Abstract

Global warming is an alarming problem in agriculture and its effect on yield loss has been estimated to be five per cent for every degree centigrade rise in temperature. Plants exhibit multiple mechanisms like optimizing signaling pathway, involvement of secondary messengers, production of biomolecules specifically in response to stress, modulation of various metabolic networks in accordance with stress, and so forth, in order to overcome abiotic stress factors. Many structural genes and networks of pathway were identified and reported in plant systems for abiotic stress tolerance. One such crucial metabolic pathway that is involved in normal physiological function and also gets modulated during stress to impart tolerance is polyamine metabolic pathway. Besides the role of structural genes, it is also important to know the mechanism by which these structural genes are regulated during stress. Present review highlights polyamine biosynthesis, catabolism, and its role in abiotic stress tolerance with special reference to plant systems. Additionally, a system based approach is discussed as a potential strategy to dissect the existing variation in crop species in unraveling the interacting regulatory components/genetic determinants related to PAs mediated abiotic stress tolerance.

## 1. Introduction

Global warming has emerged as major environmental challenge that modulates diverse environmental factors like temperature extremities, altered oxygen levels, and salt and mineral deficiency including toxicity. In bird's eye view, global warming is being visualized as climate change that transmits cautious signals for living organisms to modulate themselves so as to withstand the environmental effects. Impact of climate change on plant system, expressed in multitude of forms like drought, heat, oxidative burst, salinity, and so forth, has shifted the focus of plant biotechnology more towards dissection of genetic elements involved in stress tolerance [1]. It has been estimated that there is a potential yield loss of up to five per cent for rise in temperature by every degree centigrade [2]. Environmental changes are the signals, perceived by plant sensors and transmitted through secondary messengers to kinases which lead to altered expression of genes and metabolites through the respective transcription factors in a cascade of processes to respond and acquaint itself [3].

Bhatnagar-Mathur and coworkers [4] had classified the genes expressed under stress into three groups,* namely*, genes encoding for known enzymatic or metabolic or structural functions; proteins with unknown functions; and regulatory proteins. Irrespective of the three groups of genes that are classified based on their role during stress, it can further be grouped into two based on its applicability,* namely*, novel genes from the model organisms whose ecological habitat was at extremity and novel allelic variants of known genes that get modulated during stress period. Novel genes from model organisms will broaden our knowledge and understanding on the network of genes and metabolic pathways required to keep the organism physiologically normal even under environmental extremity. Novel allelic forms of same gene from different species resulting in differential modulation pattern and thereby effecting varied levels of stress tolerance will help to understand the biochemical and physiological mechanism that gets altered to overcome stressed conditions. Of the above said two types, in case of the former one (novel genes), it may not be just enough to transfer the genes in stress-sensitive organism to impart tolerance; rather it may require other interacting genes as well for imparting stress tolerance due to the fact that the gene itself is novel, whereas latter one (novel allelic form) will probably gain practical utility to impart stress tolerance (if the said gene is the key regulator in its metabolic pathway) in sensitive organism due to its functional expression under normal ambient conditions being known. Polyamine metabolic pathway is one such pathway in plants [5] existing under normal developmental phases (organogenesis, embryogenesis, flower and fruit development, and senescence) with a prime function to stabilize macromolecular structures [6] and gets modulated in response to various environmental stimuli of both abiotic and biotic nature [7]. This review is focused on the importance of polyamines and its biosynthetic pathway in imparting abiotic stress tolerance with special reference to crop plants.

## 2. Polyamine Biosynthesis in Plants

**Figure 1:**
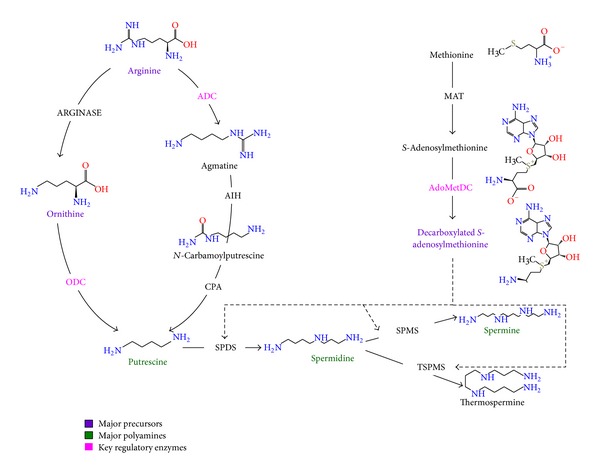
Polyamine biosynthetic pathway with special reference to plants.* ADC*: arginine decarboxylase (EC 4.1.1.9);* AdoMetDC*:* S*-adenosylmethionine decarboxylase (EC 4.1.4.50);* AIH*: agmatine iminohydrolase (EC 3.5.3.12);* CPA*:* N*-carbamoylputrescine amidohydrolase (EC 3.5.1.53);* MAT*:* S*-adenosylmethionine synthetase or methionine adenosyltransferase (EC 2.5.1.6);* ODC*: ornithine decarboxylase (EC 4.1.1.17);* SPDS*: spermidine synthase (EC 2.5.1.16);* SPMS*: spermine synthase (EC 2.5.1.22); and* TSPMS*: thermospermine synthase (EC 2.5.1.79).

Polyamines, cationic compounds having two or more amine groups, are low molecular weight organic molecules present in most of the organisms with diverse functions due to the diversity in number and position of amino groups [8]. Due to the cationic nature of polyamines, they bind easily with DNA, RNA, and proteins through electrostatic linkages resulting either in stabilization or destabilization [9, 10]. Polyamines are known to be involved in various developmental processes,* namely*, survival of plant embryos and translation in eukaryotes [11]; cell signaling and membrane stabilization [12]; cell proliferation and modulated gene expression [13]; and apoptosis and cell death [14, 15]. Putrescine (Put), spermidine (Spd), and spermine (Spm) are the major polyamines found in higher plants either in free or soluble conjugated (mainly in the form of hydroxycinnamic acid amides) or insoluble bound forms [5, 16]. Besides some unique polyamines like caldopentamine and caldohexamine that are specific to certain organisms with special reference to thermophiles including thermospermine, a structural isomer of spermine [17]. Numerous reviews on metabolic pathway with genes and enzymes involved in polyamine biosynthesis were available [18–20] and an overview was provided in Figure 1.

Occurrence of putrescine was detected in ergot as early as 1908 [21] and in potassium deficient barley plants [22]. Formation of putrescine from arginine through ornithine in the presence of ARGINASE (EC 3.5.3.1) was reported [23]. Within few years, Nakamura in 1944 had identified an alternate pathway for putrescine from arginine through agmatine which shows that in bacteria arginine is the primary precursor [24]. The difference between the two pathways is that agmatine is formed through decarboxylation reaction whereas, in the former pathway, after ornithine formation decarboxylation reaction takes place. With special reference to plants, Coleman and Hegartv in 1957 had used radioactive ornithine (^14^C) in barley for formation of putrescine [25], whereas Smith and Richards in 1962 had reported the formation of radioactive putrescine through radioactive arginine (^14^C) feeding experiments in barley [26]. These two experiments clarify the possibility of two independent pathways one from arginine and another from ornithine for putrescine biosynthesis. Greene in 1957 had showed in* Neurospora crassa* that methionine and ATP are required for the formation of spermidine and spermine by using 2-C^14^-DL-methionine [27]. Experiments by Tabor and coworkers in* Escherichia coli* had confirmed that putrescine is the precursor for spermidine and spermine biosynthesis by using C^14^-N^15^-putrescine [28]. With these studies, overall picture on biosynthetic pathway of standard polyamines,* namely*, putrescine, spermine, and spermidine, was elucidated. Ornithine or arginine acts as a primary precursor for putrescine in polyamine biosynthesis through ornithine decarboxylase (ODC; EC 4.1.1.17) or arginine decarboxylase (ADC; EC 4.1.1.9), agmatine iminohydrolase (AIH; EC 3.5.3.12), and N-carbamoylputrescine amidohydrolase (CPA; EC 3.5.1.53), respectively, with special reference to plants [10]. Decarboxylated* S*-adenosylmethionine (dcSAM) is the key aminopropyl group donor for synthesis of spermidine from putrescine through spermidine synthase (SPDS; EC 2.5.1.16); spermine from spermidine through spermine synthase (SPMS; EC 2.5.1.22); and thermospermine (tSpm), a structural isomer of spermine, from spermidine through thermospermine synthase (ACL5 or TSPMS; EC 2.5.1.79). dcSAM is synthesized by action of* S*-adenosylmethionine decarboxylase (AdoMetDC; EC 4.1.4.50) on* S*-adenosyl-methionine which in turn is synthesized through* S*-adenosylmethionine synthetase or methionine adenosyltransferase (MAT; EC 2.5.1.6) from methionine [29]. Of all these genes involved in polyamine biosynthesis, key regulators (with reference to plant system) in the polyamine biosynthesis,* namely*,* Odc*,* Adc*, and* AdoMetDC* genes, were known to act as a key regulator in modulating the endogenous levels of polyamines (Put, Spd, and Spm) during various developmental stages and including stressed conditions [30].

## 3. Polyamine Catabolism in Plants

Endogenous titers of polyamines (especially Put, Spd, and Spm) are modulated not only through regulated gene expression patterns of polyamine biosynthetic genes but also through the regulated expression of genes involved in catabolism of polyamines (generally known as amine oxidases) that catalyze the oxidative deamination of polyamines having its own functionalities. In other ways, we can even call it biosynthetic pathway of H_2_O_2_ because, in plants, compartmentalized production of H_2_O_2_ through oxidation of polyamines was reported to have functional input in cell wall maturation and lignifications during ontogeny [31–33] and also help in combating biotic [29, 34–36] and abiotic stresses [29, 37–39].

Amine oxidases, which catalyze oxidation of polyamines through deamination, are of two types,* namely*, copper dependent amine oxidase (CuAO; EC 1.4.3.6) and the flavin dependent polyamine oxidase (PAO; EC 1.5.3.11) that help in polyamine homeostasis spatially and temporally [31]. CuAO oxidizes diamines,* namely*, putrescine (Put) and cadaverine (Cad) at primary amino group, and PAO oxidizes Spd and Spm along with their acetylated derivatives at the secondary amino group [40]. Oxidation of putrescine by CuAO in plants results in formation of ammonia, H_2_O_2_, and 4-aminobutanal. 4-Aminobutanal and Δ^1^-pyrroline are interconverted spontaneously and by the catalytic action of aminobutyraldehyde dehydrogenase (ABALDH; EC 1.2.1.19;* synonym*: 1-pyrroline dehydrogenase) on Δ^1^-pyrroline, *γ*-aminobutyric acid (GABA) is produced [31] and upon its transamination and oxidation yields succinic acid which enters Krebs cycle [41]. PAOs in plant system oxidize the carbon at the* endo*-side of the N^4^-nitrogen of Spd and Spm resulting in the production of 4-aminobutanal and* N*-(3-aminopropyl)-4-aminobutanal, respectively, with H_2_O_2_ and 1,3-diaminopropane in common thereby leading to the terminal catabolism of polyamines [42]. Many uncommon polyamines like caldopentamine, caldohexamine, homocaldopentamine, homocaldohexamine, norspermidine, and norspermine were produced through the involvement of PAO and known to be involved in ontogeny of various organisms under environmental extremities [43].

Compartmentalization of substrates (polyamines) might play a major role in regulating its catabolic pathway for specific destined physiological processes like lignification, oxidative burst, suberization, and defense mechanisms, as PAO and CuAO are present extracellularly [37, 41]. Tavladoraki and coworkers in 2006 had reported for the first time in plant system the polyamine back-conversion pathway by AtPAO1 through heterologous expression [44], which recent reports also authenticate [45–47]. In case of back-conversion pathway in animal system, spermidine and spermine were acetylated through SSAT (spermidine/spermine* N* ^1^-acetyltransferase; EC 2.3.1.57) by using acetyl-CoA and this step was reported to be the rate limiting step of catabolic pathway [48]. So far in plant system, back-conversion of spermine by AtPAO1 was reported to be specific for spermine and occur independently of SSAT, the acetylation step, as in case of animal system [44], though there were reports on the presence of glutamyl and acetyl derivatives of polyamines in chloroplasts [49] and on the presence of back-conversion pathway (irrespective of SSAT-dependent or -independent) using ^14^C studies [50, 51]. Rather, spermidine acts as a competitive inhibitor for the conversion of spermine in the presence of AtPAO1 [44]. Activity of AtPAO1 was much lower in conversion efficiency while using* N* ^1^-acetylspermine as substrate [44]. Though back-conversion of polyamines in both plant and animal system has been reported, question still remains about the importance of acetylation in back-conversion process with special reference to animal system (in evolutionary view point in comparison with plant system), since acetylation is a prerequisite for back conversion only in animal system [40, 44]. Also, comparison of different plant PAOs (monocot and dicot) with its corresponding counterparts from animal system may help in identifying the actual genetic variation or causal factor for the specificity of back-conversion process only for spermine and not spermidine in plant system.

## 4. Polyamines and Abiotic Stress

Polyamines modulate the plant's response to much broader range of abiotic stresses than expected,* namely*, drought, salinity, heavy metal toxicity, oxidative stress, chilling injury, high temperature, osmotic stress, water logging, and flooding tolerance as proved either by exogenous application of polyamines or by development of transgenic plants overexpressing the genes involved in polyamine biosynthesis [52]. Modulated levels of polyamines may act either as a signal or as a messenger (to transmit the perceived signals from the sensors) to articulate the plants' behavioral response spatially and temporally in order to avoid or overcome stress. Altered endogenous polyamine (free or conjugated or bound) levels are known to be involved in formation of polyamine-RNA complexes, thereby generating structural changes in RNA (m-, r-, and t-) at physiological concentrations of potassium and magnesium ions [13]. Covalent linkage of polyamines to various enzymes or proteins (posttranslational modification) involved in physiological processes under normal or stressed conditions was catalyzed by transglutaminase (TGase; EC 2.3.2.13) class of enzymes [53, 54].

**Figure 2:**
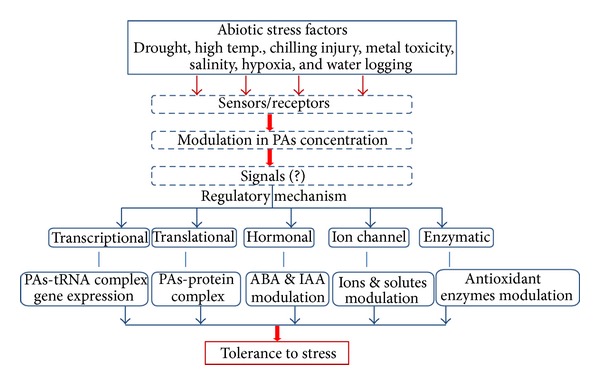
Overview of polyamines (PAs) mediated abiotic stress tolerance in plants.

Of various abiotic environmental stimuli under which polyamines get modulated and thereby its cellular functions were mineral nutrient deficiency [22, 25], metal toxicity [55, 56], salinity [16, 30], high [17] and low temperature [30], drought [4, 6], hypoxia [38], osmotic [16], and oxidative factors [38, 52, 57]. Polyamines, besides responding to external stimuli by their modulated titers, also alter ion channels [11]; stimulate special kind of protein synthesis; stimulate assembly of 30S ribosomal subunits; and stimulate Ile-tRNA formation [13]. Also, modulated titers of polyamines in combination with epibrassinolides, active form of brassinosteroids, were reported to regulate abscisic acid (ABA) and indole-3-acetic acid (IAA) pathways which in turn enhances tolerance to metal toxicity [55]. Besides heavy metals themselves being toxic to plants, they also stimulate oxidative stress due to the fact that heavy metals are basically ionic in nature. Polyamines in combination with brassinosteroids besides modulating ABA and IAA pathways with their cascading effects for heavy metal tolerance also modulate levels of antioxidants like glutathione, ascorbic acid, proline, glycine-betaine, and so forth and antioxidant enzymes like glutathione reductase, superoxide dismutase, catalase, peroxidase, and so forth to impart stress tolerance [56]. Brief overview of polyamines on abiotic stress tolerance was provided in Figure 2. Enhanced levels of polyamines either through exogenous feeding [52] or through heterologous expression of polyamine biosynthetic genes in transgenic plants [58] had been shown to enhance abiotic stress tolerance. However, use of constitutively expressed promoters like CaMV35S, ubiquitin, and actin with polyamine biosynthetic genes towards stress tolerance may produce modulated polyamine levels even under normal conditions resulting in deleterious effects and thereby reducing yield which is a special concern towards agricultural crops [59].

## 5. Molecular-Genetic Regulation of Polyamines (PAs)

In plants greater accumulation of PAs (Put, Spm, and Spd) during abiotic stress is well documented and is implicated in increased tolerance to abiotic stress [60]. Induction of majority of genes associated with PAs biosynthesis during one or another type of abiotic stresses suggests the plausible functional relation between PAs metabolism and abiotic stress factors [39, 61]. Moreover induction of PAs biosynthetic genes by ABA, a known regulator of stress which acts upstream of PAs biosynthetic pathway [62]; changes in cellular level of PAs during abiotic stress condition [63]; and enhancement of abiotic stress tolerance in many plant species upon exogenous application of PAs [64] all support the strong connection between PAs and stress.

Though a clear picture of roles of PAs in abiotic stress is beginning to emerge, their unequivocal role in imparting abiotic stress tolerance is puzzled/complicated by the fact that substrate of PAs is also shared by other important metabolites such as ethylene, proline, NO, and metabolites associated with N-metabolism [61] as well. The silencing of ethylene biosynthetic genes ACC synthase and ACC oxidase with concomitant increase in PAs (Put and Spd) leads to improved abiotic tolerance in tobacco plant [65]. Also, modulation of arginase (an enzyme of N-metabolism) resulted in alteration of PAs (Put and Spm), thus effecting enhancement of abiotic stress tolerance [66]. Additionally, modulation of PAO, the enzyme associated with PAs catabolism, resulted in salt stress tolerance in tobacco [67].

In addition to the above, the combinatorial network of action among the ABA, NO, and PAs vis-a-vis abiotic stress has also started to unfold the interactomes associated with regulation of abiotic stress tolerance. The observation of ABA induced accumulation of PAs [68] and induction of NO synthesis in different plant species through PAs (Spd and Spm) catabolism by CuAO and PAO [29, 69] during abiotic stress reveals the complex interaction among these three metabolites, namely, ABA, Pas, and NO. Moreover the cellular balance of PAs in cell is mainly determined through the regulation of its biosynthesis and catabolism. In plants the PAs concentrations are much higher than those of phytohormones; however plant PAs are also regarded as growth regulators due to their diverse role during the course of plant growth and development [70].

Among the factors which modulate cellular concentration of PAs, it is the species nature and their degree of tolerance and sensitivity towards stress are of paramount importance for gaining knowledge and translating such idea into agriculturally important crop species [71]. In this regard, there is lack of sufficient information as how the levels of PAs are being modulated among the allelic variants of PAs and interacting metabolites biosynthetic genes found among natural population of economically important crop species. There are numerous reports on differential expression of genes due to allelic variation within a species [72]. Also, there are reports on altered levels of expression of the same gene in different species [73], thereby exhibiting enormous diversity in expression efficiency. These reports indicate variability at nucleotide level within or between species resulting in altered gene expression levels due to various environmental factors or spatial and temporal expressions. The heterologous expression of* DsAdoMetDC* [74] and* DsAdc* [75] gene from* Datura stramonium* in rice resulted in robust recovery from drought in transgenic rice plants despite having endogenous* AdoMetDC* and* Adc* gene. This indicates that possible sequence variation in genic region regulates expression levels, thereby modulating the efficiency and activity of the enzymes involved in polyamine biosynthesis.

In spite of the huge genetic variation existing in plants, identification and utilization of allelic variation within the species to enhance stress tolerance are still a major area of research that needs a conscious effort. The knowledge gained from genomics assisted discovery of biosynthetic genes of PAs and its signaling pathway in model plant Arabidopsis [68] can well be translated into agriculturally important crops to generate stress tolerant plants [60]. Analyses of variants under natural rather than subjected to controlled condition have proved quite useful in dissecting the novel allele in Arabidopsis as subtle variations in gene expression between individuals are largely unmasked and thus are quite useful approach to uncover functional links between genes and unravel regulatory influences [76]. The above aspect assumes significance as finding a suitable variant tolerant to stress can act as donor through either molecular breeding or genetic engineering approaches for genetic enhancement of stress tolerant traits in targeted crop species.

**Figure 3:**
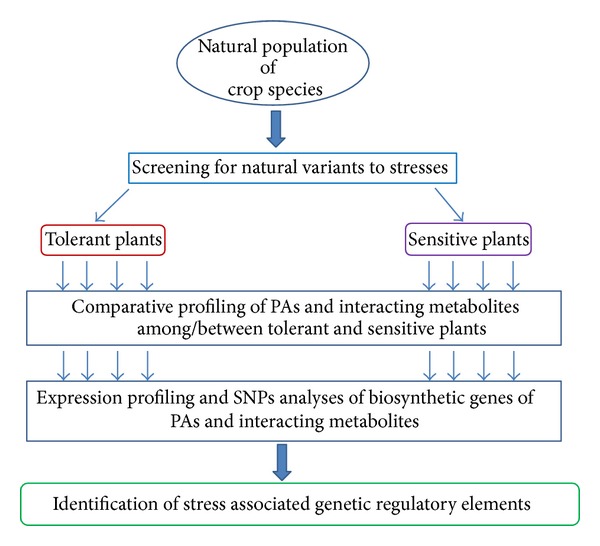
Schematic representation of system based analyses for identification of abiotic stress tolerance regulatory genetic elements.

With the present state of knowledge of PAs mediated enhancement of stress tolerance as demonstrated from molecular-genetic studies (loss or gain of function mutants) and overexpression studies involving biotechnological tools in different plant species, it has become relatively feasible to screen and identify the natural variant within existing collection of plants of a particular crop species. The screening of collection of putative stress tolerant plants for PAs and interacting metabolite profiling using GC-MS, LC-MS, and other appropriate tools can be quite useful for gaining knowledge of overall status of metabolites. Such “targeted” metabolomic studies are quite useful in deciphering function of genes in system based approach [77]. Simultaneously, the genomics approach using expression profiling and SNPs assay of potential genes can further elaborate the functional correlation between metabolite and genes associated with PAs mediated stress tolerance. A brief overview of schematic for such system based analyses is presented (Figure 3). Moreover, integration of both metabolomics and genomics data not only reveals the complex molecular regulation of PAs mediated stress tolerance but also demonstrates how such physiological phenomena are being regulated by metabolites and their fluxes.

## 6. Conclusion and Future Perspectives

It is possible to assign the functionality to genes/sequences, but, it is much difficult to understand its behavior spatially and temporally in a systems approach manner under different environmental conditions at species level or even at genotype level. It is mainly due to the altered expression levels for same gene in different species [73] or even due to allelic variation existing between different genotypes within a species or even within a genotype [72]. Recently, understanding about polyamines in response to stress condition in plants had increased significantly. However, to understand the interconnections of PAs biosynthesis, its catabolism, and conjugation along with PAs signaling aspect a detailed metabolite profiling among allelic variants will provide a framework for unraveling genetic determinants (structural/regulatory) with genomic assisted analyses. Additionally, the novel regulatory mechanisms based on small regulatory RNA [78] and uORF [79] can further accelerate our effort to provide clues in reasoning the mysteries involved in basic (back-conversion specificity for spermine in plant system) and applied (modulating polyamines to impart stress tolerance) sciences towards better understanding of polyamine metabolism. Emphasis on the above approaches will provide us with appropriate genomic tools to manage abiotic stress, the immediate effect of climate change, in plant systems with special focus on crop plants. Therefore, with the existence of natural variation, it will be an excellent opportunity to dissect the existing genetic diversity for identifying genotypes possessing compatible allelic variants for imparting stress tolerance in an efficient manner.
